# Recombinant growth hormone therapy in children with Turner Syndrome in Korea: a phase III Randomized Trial

**DOI:** 10.1186/s12902-021-00904-5

**Published:** 2021-12-10

**Authors:** Jinsup Kim, Min-Sun Kim, Byung-Kyu Suh, Cheol Woo Ko, Kee-Hyoung Lee, Han-Wook Yoo, Choong Ho Shin, Jin Soon Hwang, Ho-Seong Kim, Woo Yeong Chung, Chan Jong Kim, Heon-Seok Han, Dong-Kyu Jin

**Affiliations:** 1 Department of Pediatrics, Hanyang University Medical Center, Hanyang University College of Medicine, Seoul, South Korea; 2 Department of Pediatrics, Samsung Medical Center, Sungkyunkwan University School of Medicine, 81 Irwon-Ro Gangnam-gu, Seoul, 06351 South Korea; 3 Department of Pediatrics, Seoul St. Mary’s Hospital, The Catholic University of Korea, Seoul, South Korea; 4 Department of Pediatrics, Kyungpook National University Hospital, Daegu, Republic of Korea; 5 Department of Pediatrics, Korea University Anam Hospital, Seoul, South Korea; 6 Department of Pediatrics, Medical Genetics Clinic and Laboratory, Asan Medical Center Children’s Hospital, University of Ulsan College of Medicine, Seoul, South Korea; 7 Department of Pediatrics, Seoul National University Children’s Hospital, Seoul, South Korea; 8 Department of Pediatrics, Ajou University Hospital, Suwon, South Korea; 9 Department of Pediatrics, Severance Hospital, Yonsei University College of Medicine, Seoul, South Korea; 10 Department of Pediatrics, Inje University Busan Paik Hospital, Busan, South Korea; 11 Department of Pediatrics, Chonnam National University Hospital, Gwangju, South Korea; 12 Department of Pediatrics, Chungbuk National University Hospital, Cheongju, South Korea

**Keywords:** Short stature, Turner syndrome, Growth hormone

## Abstract

**Background:**

Short stature is the most consistent characteristic feature of Turner syndrome (TS). To improve final heights of children with TS effectively, it is important to provide them with early and appropriate treatment using growth hormone (GH). The objective of this study was to assess the efficacy and safety of a new recombinant human GH, Growtropin®-II (DA-3002, Dong-A ST Co., Ltd) versus a comparator (Genotropin®, Pfizer Inc.) for Korean children with TS.

**Methods:**

This open-label, active-controlled, parallel-group, randomized controlled phase III trial was conducted at 11 hospitals in Korea. Eligible patients (*n* = 58) were randomized to two groups: 1) DA-3002 group (administrated with DA-3002 at 0.14 IU [0.0450–0.050 mg] /kg/day); and 2) comparator group (administrated with the comparator at 0.14 IU [0.0450–0.050 mg] /kg/day).

**Results:**

The change from baseline in annualized height velocity (HV) after a 52-week treatment period was 4.15 ± 0.30 cm/year in the DA-3002 group and 4.34 ± 0.29 cm/year in the comparator group. The lower bound of 95% two-sided confidence interval for group difference in the change of annualized HV (− 1.02) satisfied the non-inferiority margin (− 1.5). The change in height standard deviation score (HtSDS) at 52-week was 0.70 ± 0.23 for the DA-3002 group and 0.66 ± 0.39 for the comparator group, showing no significant (*p* = 0.685) difference between the two groups. The change of skeletal maturity defined as change in bone age/change in chronological age between the two groups was not significantly different (1.25 ± 0.58 for the DA-3002 group and 1.47 ± 0.45 for the comparator group, *p* = 0.134). Changes from baseline in serum insulin-like growth factor-1 (IGF-1) and insulin-like growth factor binding protein-3 (IGFBP-3) after 52 weeks of treatment did not differ significantly between the two groups (*p* = 0.565 and *p* = 0.388, respectively) either. The occurrence of adverse events was not statistically different between groups.

**Conclusions:**

This study demonstrates that the efficacy and safety of GH treatment with DA-3002 in children with TS are comparable with those of the comparator. It is expected to analysis the long-term effect of DA-3002 on the increase of final adult height in children with TS and possible late-onset complications in the future.

**Trial registration:**

The study was registered at ClinicalTrials.gov. ClinicalTrials.gov identifier: NCT01813630 (19/03/2013).

## Background

Turner syndrome (TS) is related to a defect in all or part of one X-chromosome that is missing or structurally altered. It occurs in one of 2500–3000 live-born females [1]. TS is characterized by short stature, dysmorphic features, loss of ovarian function, and cardiac abnormalities [2]. Among them, short stature is the most consistent characteristic feature of TS. Untreated girls with TS achieve an average adult height of ~ 140 cm [3, 4]. The growth rate of girls with TS is often decreased in the first 3 years of life. More than half of girls with TS will fall below the fifth percentile by 2 years of age [1, 5]. Failure to experience a pubertal growth spurt because of gonadal dysgenesis also contributes to a typical short stature.

Treatment with recombinant human growth hormone (rhGH) is a standard care for a girl with TS. It has been approved by the US Food and Drug Administration since 1997 [6]. Growth hormone (GH) therapy is usually started as early as 2 years of life after a girl’s height is found to be below the fifth percentile for a healthy population in the same age cohort. Many studies have evaluated GH therapy in girls with TS and revealed that GH can increase adult height by 5–12 cm with good safety [6–10]. Longer duration of GH treatment with higher dose at earlier age showed further increase of final adult height [6, 11]. Earlier GH treatment to normalize stature before the onset of puberty can alleviate physical and psychosocial problems [6]. However, there was no established optimal age to start GH treatment that increase final adult height. Recent study showed that early initiation of GH by age 6 years without inconsistencies of treatment enable attainment of final adult height within normal range in girls with TS regardless of whether started during the toddler stage or not [12]. Childhood GH treatment also has beneficial effect on lipid profile and cardiovascular diseases in women with TS [13].

The present study was a one-year, open-label, multi-center, randomized controlled trial (RCT) of recombinant human GH administered to Korean girls with TS. The primary purpose of this study was to assess the efficacy and safety of daily subcutaneous rhGH, Growtropin®-II (DA-3002, Dong-A ST Co., Ltd., Seoul, Korea) and compare effects between Growtropin®-II and Genotropin® (Pfizer Inc., New York, USA) in Korean children with TS. DA-3002 is a rhGH of liquid formulation that administered via a pen. Comprehensive efficacy and safety data of GH therapy are provided here.

## Methods

### Subjects

The following subjects were included in this study: (1) prepubertal children who were diagnosed with Turner syndrome through a chromosome test; (2) children with chronological age of 2 years to 12 years; (3) children whose annualized height velocity (HV) was less than 6 cm with bone age of 12 years or younger and height in the 10th percentile or less among Korean population of the same chronological age prior to the participation in the study;(11) (4) pre-adolescent children at Tanner stage I (Breast); (5) children with normal range of HbA1c level and normal thyroid function (possible even if the function was normal through hormone therapy); (6) children who had a record (e.g., hospital record, school health record, etc.) of official height at least 6 months before the start of this study (self-measurement record was not accepted) and who had not received an agent (rhGH, androgen, estrogen) that could affect growth after the height was recorded; (7) the child and parent (or legal custodian or legal guardian) signed a written consent form after listening to the explanation of this clinical study.

Exclusion criteria were as follows: (1) children who had received growth hormones for 12 months or more in the past; (2) children who received estrogen for more than two months from outside in the fetal period; (3) children who had been treated with estrogen or adrenal androgens for 12 months or more in the past; (4) children who had Y component in the chromosome analysis and reproductive tissue (or gonads) not previously removed; (5) children who had clinically significant congenital or acute/chronic disease, including endocrine and metabolic diseases (e.g., diabetes, diabetic retinopathy, etc.), malignant tumor, central nervous system trauma (CNS Trauma), psychiatric disorder, active chronic infections (e.g., tuberculosis, etc.), acute respiratory failure, hypersensitivity to growth hormone preparations, and dwarfism due to brain tumor; (6) children taking amphetamines or other drugs that might interfere with growth hormone secretion or action (e.g., methylphenidate, pemoline, corticosteroid, estrogen, androgen); (7) children with a closed osteoepiphysis; (8) children who participated in other clinical studies within three months before the start of this study; (9) children who were evaluated as inappropriate by the clinical study investigator.

## Methods

This open-label, active-controlled, parallel-group, randomized controlled phase III study was conducted at 11 hospitals in Korea from February 2013 to May 2018. This study was conducted in compliance with ethical guidelines of the Declaration of Helsinki and Good Clinical Practices after obtaining approval from the institutional review board of each study site. Written informed consent was obtained from all participants and legally authorized representatives of patients since all participants were under 18 years of age. The study was registered at ClinicalTrials.gov. (ClinicalTrials.gov identifier: NCT01813630, date of registration: 19/03/2013).

**Fig. 1:**
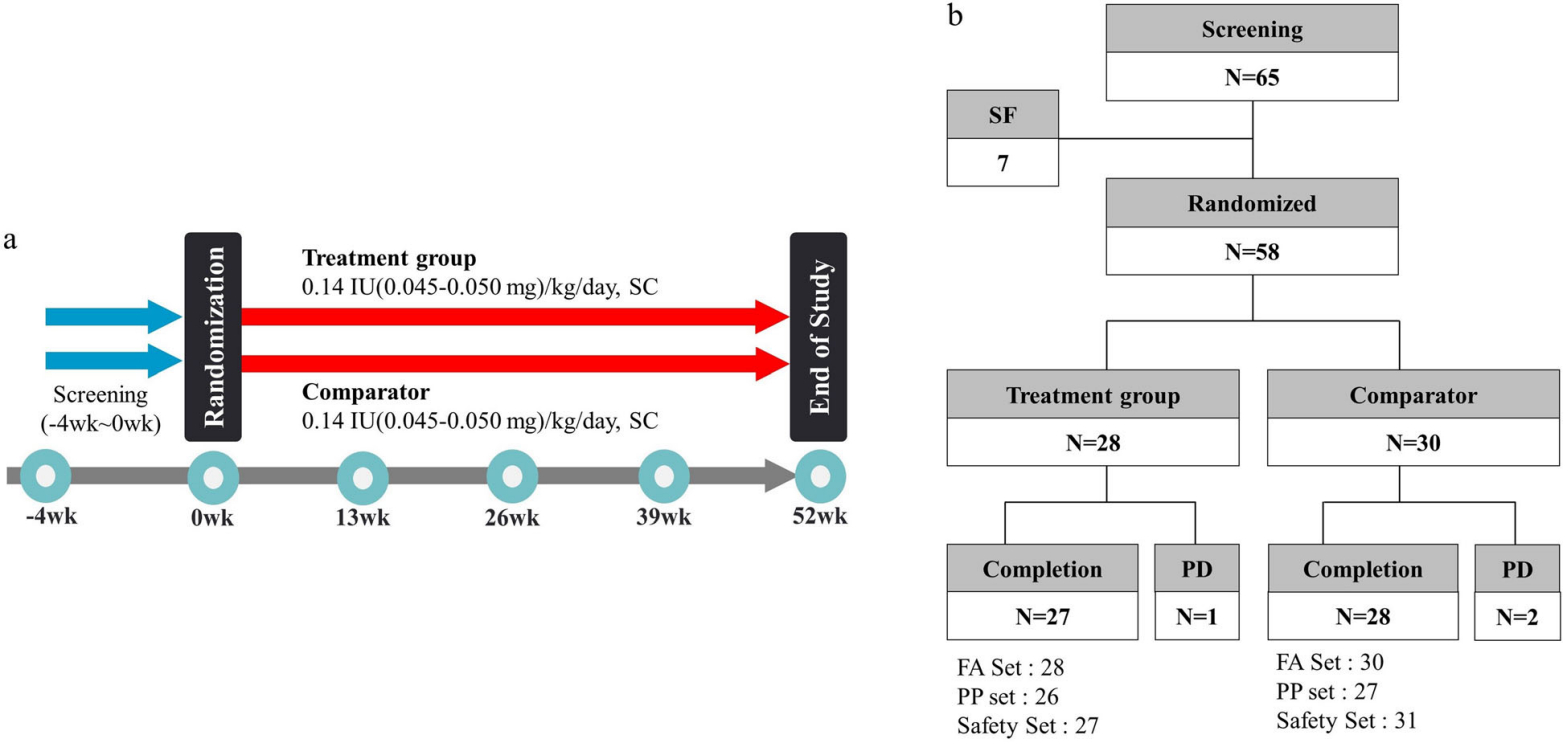
Study design and disposition. **a** study design; **b** study disposition. SF: Screening failure, PD: Premature discontinuation

Through screening tests (− 4 weeks to 0 weeks), subjects who were eligible based on inclusion and exclusion criteria were randomized to a treatment group or a comparator group. The treatment group received DA-3002 while the comparator group received Genotropin® at a dose of 0.14 IU (0.045–0.050 mg)/kg/day by subcutaneous injection for 52 weeks (Fig. 1). Each subject visited the institution at 13, 26, 39, and 52 weeks for measurements of height, weight, insulin-like growth factor-1 (IGF-1) and insulin-like growth factor binding protein-3 (IGFBP-3) levels, thyroid function, hemoglobin A1c level, and other laboratory tests. Subjects also responded to a questionnaire on drug compliance and adverse events in person during visits of the institution or by telephone at the mid time point of each visit (6, 19, 32, and 45 weeks).

### Measurements

The primary efficacy endpoint was change from baseline in annualized HV at 52 weeks between treatment and comparator groups. Secondary efficacy endpoints were change from baseline in height standard deviation score (HtSDS) at 52 weeks, changes of skeletal maturity at 26 and 52 weeks (changes in bone age/changes in chronological age) and changes from baseline in IGF-1 and IGFBP-3 levels at 26 and 52 weeks. Safety assessment consisted of clinical laboratory findings, growth hormone antibody formation, monitoring of vital signs, abnormal physical findings, and adverse events such as subjective and objective symptoms (including observation of local irritant reaction).

### Statistics

Statistical analysis was performed using SAS statistical software ver. 9.4 (SAS Institute, Cary, NC, USA). Results of descriptive statistics are presented as mean ± standard deviation (SD) for continuous variables and frequency or percentage for categorical variables. The primary efficacy endpoint was analyzed using the ANCOVA (Analysis of Covariance) model with treatment group as an effect and chronological age at baseline as a covariate. When the lower limit of 95% two-sided confidence interval (i.e., a 97.5% one-sided confidence interval) for the difference between the treatment group (DA-3002) and the comparator group (Genotropin®) in the ANCOVA model was greater than or equal to − 1.5, the treatment group was judged to be non-inferior to the comparator group. The secondary efficacy endpoint was analyzed using a two-sample t-test or the Wilcoxon rank sum test to assess differences between the treatment group and the comparator group. The number and incidence rate (%) of subjects who experienced Treatment-Emergent Adverse Events (TEAEs), adverse drug reactions (ADRs), and serious adverse Events (SAEs) were presented. The Chi-square test or Fisher’s exact test was used to determine whether there was a significant difference between groups. A *p*-value of less than 0.05 was considered statistically significant.

## Results

### Subjects’ characteristics and disposition

A total of 65 subjects with TS were screened from 11 hospitals. Of them, 58 were enrolled and randomly assigned to the treatment group (DA-3002, *n* = 28) or the comparator group (Genotropin®, *n* = 30). The distribution of subjects is shown in Fig. 1. There were seven children with screening failure due to withdrawal of consent in two subjects, violation of inclusion criteria in four subjects, and compliance with exclusion criteria in one subject. Karyotypes and clinical characteristics of study subjects with TS are summarized in Table 1. Only four subjects had a 45,X karyotype. More than half (*n* = 43 [74.1%]) of subjects were revealed to have mosaicism. Structural abnormalities of the X chromosome without mosaicism were found in 11 (19.0%) girls with TS. The proportion of particular karyotypes was comparable between treatment and comparator groups. The mean (±SD) age of subjects was 6.84 ± 2.62 years for the treatment group and 7.06 ± 2.96 years for the comparator group. Other baseline characteristics were well balanced between the two groups.

**Table 1:** Karyotypes, demographics and baseline characteristics

	Treatment group (*N* = 28)	Comparator (*N* = 30)	*p*-value
Karyotype	N (%)		
Simple X monosomy (45, X)	3 (10.71)	1 (3.33)	0.345 ^b^
Structural abnormalities of the X chromosome (e.g. 46, X, i [Xq], 46, X, Xq or Xp [short or long arm deletions])	6 (21.43)	5 (16.67)	0.644 ^a^
Mosaic Karyotypes	19 (67.86)	24 (80.00)	0.291 ^a^
a. 45, X/46, XX	3 (10.71)	7 (23.33)	0.301 ^b^
b. 45, X/46, XY or 46, XY Yvar/Ydel	2 (7.14)	2 (6.67)	1.000 ^b^
c. 45, X/46, X, i (Xq) or other structural chromosome abnormality	14 (50.00)	15 (50.00)	1.000 ^a^
Baseline characteristic	Mean ± SD		
Chronological age (year)	6.84 ± 2.62	7.06 ± 2.96	0.883 ^d^
Bone Age (year)	6.03 ± 2.93	5.96 ± 2.63	0.919 ^d^
Height (cm)	106.72 ± 13.49	106.93 ± 14.91	0.955 ^c^
Height SDS	−2.36 ± 0.64	−2.44 ± 0.58	0.450 ^d^
Body Weight (kg)	20.64 ± 8.56	20.88 ± 7.31	0.686 ^d^
BMI (kg/m^2^)	17.35 ± 3.16	17.64 ± 2.46	0.339 ^d^
TSH (uIU/ml)	3.20 ± 1.64	3.01 ± 1.52	0.619 ^d^
fT4 (ng/dl)	1.40 ± 0.26	1.37 ± 0.20	0.889 ^d^
IGF-1 (ng/ml)	146.57 ± 52.20	143.15 ± 62.96	0.981 ^d^
IGFBP-3 (ug/ml)	3.95 ± 0.98	3.56 ± 0.68	0.083 ^c^

*SD* Standard Deviation, *SDS* Standard Deviation Score, *BMI* Body Mass Index, *TSH* Thyroid Stimulating Hormone, *fT4* Free Thyroxine, *IGF-1* Insulin-like Growth Factor-1, *IGFBP-3* Insulin-like Growth Factor Binding Protein-3

^a^Chi-square test between treatment groups; ^b^ Fisher’s exact test between treatment groups

^c^Two sample t-test between treatment groups; ^d^ Wilcoxon rank sum test between treatment groups

Full analysis (FA) set was defined as all subjects who received at least one dose of the study drug with at least one measurement of the primary efficacy endpoint after randomization. It consisted of a total of 58 subjects (28 in the treatment group and 30 in the comparator group). Per-protocol (PP) set was defined as all subjects who completed all 52 weeks of this study without any major protocol violation. It consisted of 53 subjects (26 in the treatment group and 27 in the comparator group). Safety set included all subjects who received at least one dose of the study drug with at least one measurement of safety-related data after dosing by telephone or visit. It had 58 subjects (27 in the treatment group and 31 in the comparator group).

### Efficacy results based on the PP set

**Fig. 2:**
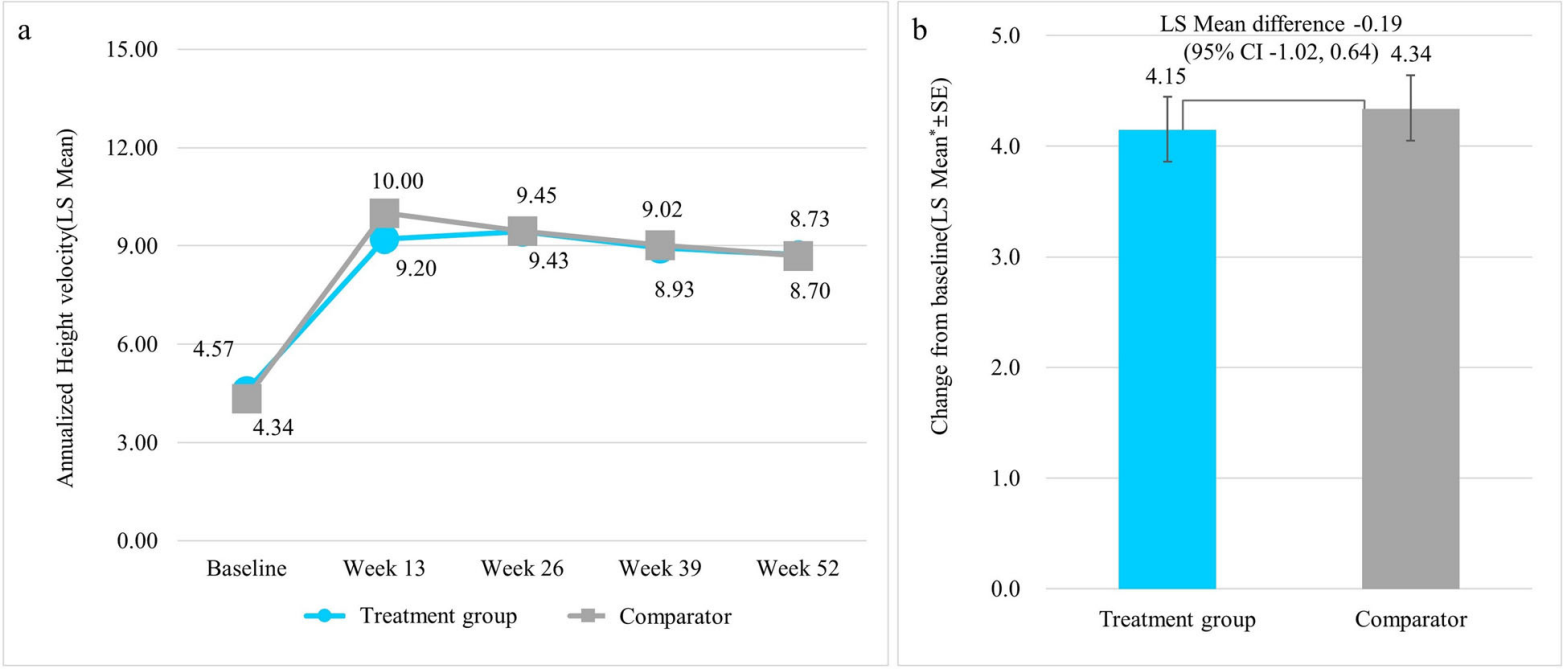
Annualized HV at baseline, 13, 26, 39, and 52-week (cm/year) and change in annualized HV from baseline at 52-week. a: Annualized HV at baseline, 13, 26, 39 and 52-week; b: Change in annualized HV from baseline at 52-week. HV: height velocity; SE: Standard Error; * LS mean difference for between-treatment groups using ANCOVA model with treatment group as a factor and CA at baseline as a covariate

The primary efficacy endpoint, change from baseline in HV at 52 weeks, was 4.15 ± 0.30 cm/year in the treatment group and 4.34 ± 0.29 cm/year in the comparator group. The difference in HV change from baseline between the two groups (treatment group - comparator group) was − 0.19 ± 0.41 cm/year [95% CI: − 1.02 to 0.64 cm/year]. The lower limit of the 95% confidence interval (i.e., 97.5% one-sided confidence interval) was − 1.02 cm/year, which was greater than the non-inferiority limit of − 1.5, proving that the treatment group was not inferior to the comparator group. In the case of changes from baseline in HV at 13, 26, and 39 weeks, both treatment and comparator groups showed statistically significant increases of HV from baseline at all time points after treatment (at 13, 26, and 39 weeks for both treatment group and comparator group, *p* < 0.001) (Fig. 2).

**Fig. 3:**
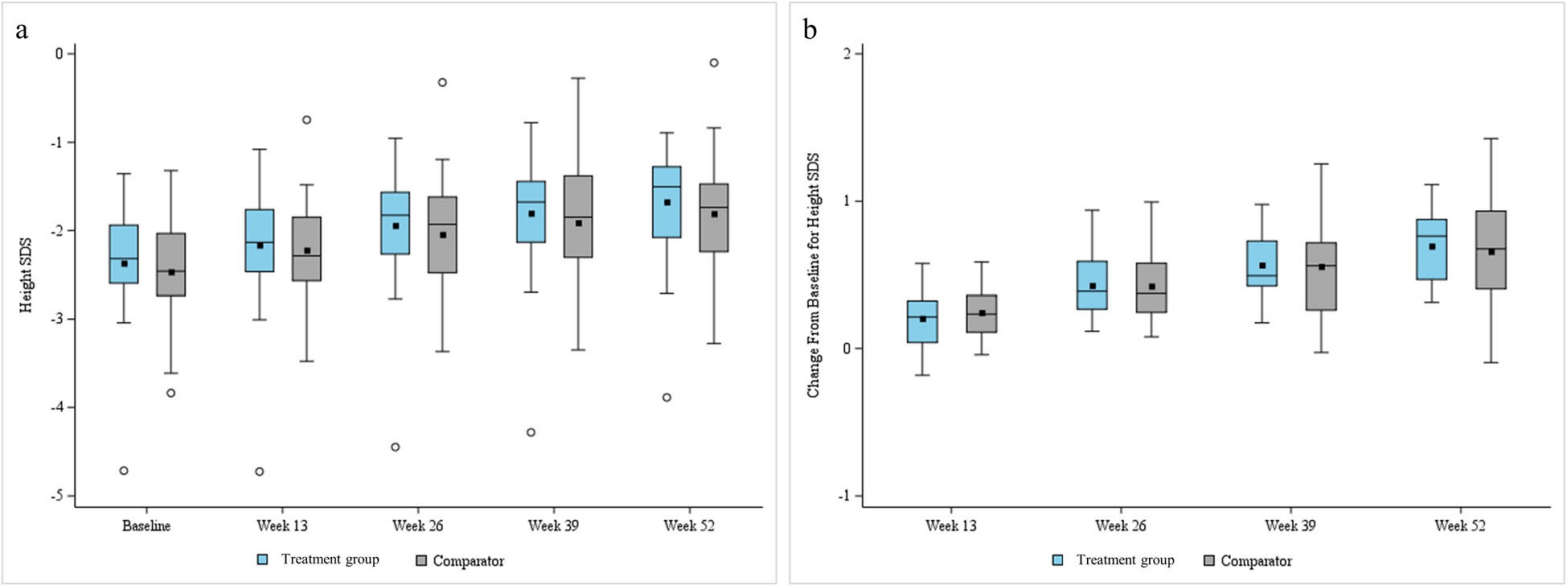
Height standard deviation scores at baseline, 13, 26, 39, and 52 weeks and change in height standard deviation score for annualized height velocity from baseline at 52-week. **a** Height standard deviation scores at baseline, 13, 26, 39, and 52 weeks; **b** Change in height standard deviation score of annualized height velocity from baseline at 52-week

The change from baseline in HtSDS was 0.43 ± 0.22 in the treatment group and 0.42 ± 0.24 in the comparator group at 26 weeks. It was 0.70 ± 0.23 in the treatment group and 0.66 ± 0.39 in the comparator group at 52 weeks (Fig. 3). Both treatment and comparator groups showed statistically significant increases from baseline in HtSDS at all time points after treatment (all *p* < 0.001). There was no statistically significant difference between the treatment group and comparator group in the change of HtSDS at any time point (at 26 weeks and 52 weeks: *p* = 0.949 and *p* = 0.685, respectively).

**Fig. 4:**
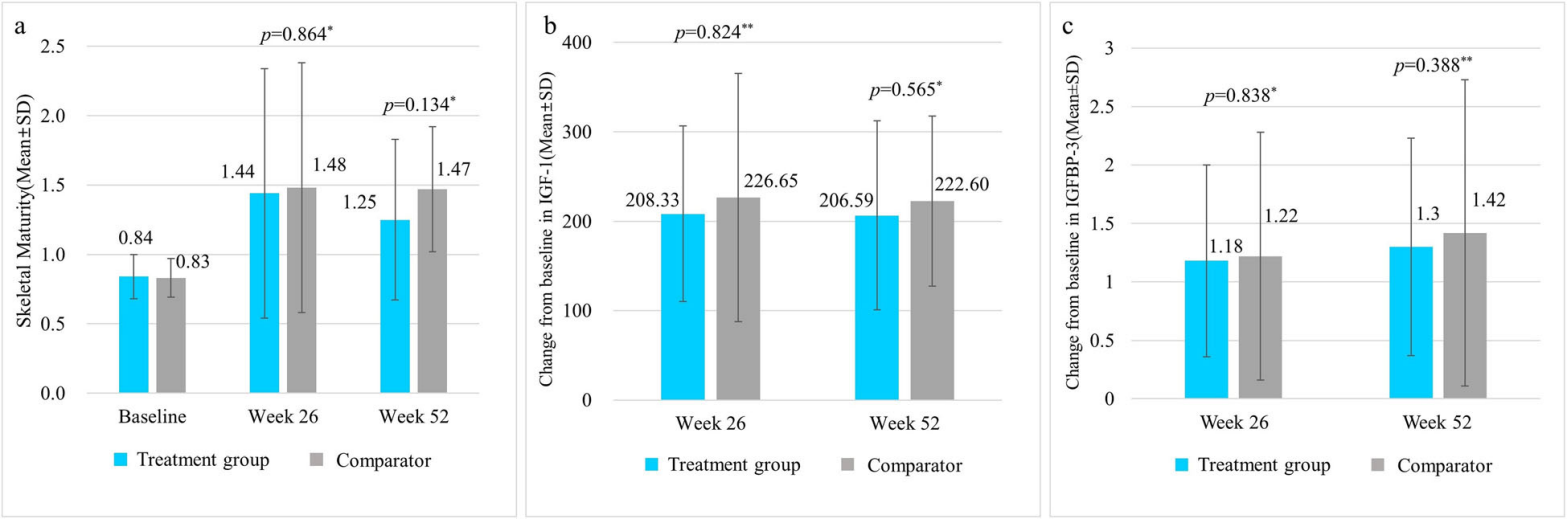
Comparison of skeletal maturity and changes in IGF-1 and IGFBP-3 levels from baseline. **a** Skeletal maturity (ratio of change in bone age/chronological age), **b** Change in IGF-1 level from baseline, c: Change in IGFBP-3 level from baseline. IGF-1: Insulin-like Growth Factor-1; IGFBP-3: Insulin-like Growth Factor Binding Protein-3. * Two sample t-test between treatment groups; **: Wilcoxon rank sum test between treatment groups

The skeletal maturity defined as change in bone age/change in the chronological age was 0.84 ± 0.16 in the treatment group and 0.83 ± 0.14 in the comparator group at baseline. It was 1.44 ± 0.90 in the treatment group and 1.48 ± 0.90 in the comparator group at 26 weeks and 1.25 ± 0.58 in the treatment group and 1.47 ± 0.45 in the comparator group at 52 weeks. Both treatment and comparator groups showed statistically significant increases from baseline in skeletal maturity at all time points after treatment (treatment group: *p* = 0.004, comparator group: *p* = 0.002 at 26 weeks; treatment group: *p* = 0.004, comparator group: *p* < 0.001 at 52 weeks). There was no statistically significant difference in the skeletal maturity between two groups at any time point (at 26 weeks and 52 weeks, *p* = 0.864 and *p* = 0.134, respectively) (Fig. 4).

In the case of changes from baseline for IGF-1 and IGFBP-3 levels, IGF-1 level change was 208.33 ± 98.22 ng/mL in the treatment group and 226.65 ± 138.93 ng/mL in the comparator group at 26 weeks. It was 206.59 ± 105.76 ng/mL in the treatment group and 222.60 ± 95.15 ng/mL in the comparator group at 52 weeks (Fig. 4). Both treatment and comparator groups showed statistically significant increases from baseline in IGF-1 levels at all time points after treatment (all *p* < 0.001). There was no statistically significant difference in IGF-1 level change between two groups at any time point (at 26 weeks and 52 weeks, *p* = 0.824 and *p* = 0.565, respectively). In the case of change in IGFBP-3 level from baseline, IGFBP-3 change was 1.18 ± 0.82 μg/mL in the treatment group and 1.22 ± 1.06 μg/mL in the comparator group at 26 weeks. It was 1.30 ± 0.93 μg/mL in the treatment group, and 1.42 ± 1.31 μg/mL in the comparator group at 52 weeks. Both treatment and comparator groups showed statistically significant increases from baseline in IGFBP-3 levels at all time points after treatment (all *p* < 0.001). There was no statistically significant difference in IGFBP-3 level change between two groups at any time point (at 26 weeks and 52 weeks, *p* = 0.838 and *p* = 0.388, respectively) (Fig. 4).

All analysis results of efficacy evaluation variables in the FA set were similar to those in the PP set.

### Safety results based on the safety set

Adverse events (TEAEs) were found in 45 subjects (77.59%): 21 subjects in the treatment group (77.78%) and 24 subjects in the comparator group (77.42%). The difference between the treatment group and the comparator group was not statistically significant (*p* = 0.974) (Table 2). The number of subjects with adverse drug reactions related to the investigational product was one (1.72%), which was one subject (3.70%) in the treatment group. No ADRs occurred in the comparator group. The incidence of ADRs showed no statistically significant difference between two groups (*p* = 0.466). Preferred term of one adverse drug reaction that occurred in the treatment group was an injection site erythema. The severity was mild. The relevance with the investigational product was not assessable.

**Table 2:** Incidence rate of Treatment-Emergent Adverse Events, Serious Adverse Events and Adverse Drug Reactions

	Treatment group (*N* = 27)	Comparator (*N* = 31)	*p*-value
TEAEs [N, %]	[21, 77.78]	[24, 77.42]	0.974^a^
SAEs [N, %]	[3, 11.11]	[3, 9.68]	1.000^b^
ADRs [N, %]	[1, 3.70]	[0, 0.00]	0.466^b^

*TEAE* Adverse event, *SAE* Serious Adverse Event, *ADR* Adverse Drug Reactions

^a^Chi-square test between treatment groups; ^b^Fisher’s exact test between treatment groups

Serious adverse events occurred in 6 subjects (10.34%) in this clinical study: 3 subjects (11.11%) in the treatment group and 3 subjects (9.68%) in the comparator group. There was no statistically significant difference between the treatment group and the comparator group (*p* = 1.000). There were no serious adverse drug reactions, adverse events that resulted in permanent discontinuation of the investigational product, or adverse events that resulted in death during this clinical study.

Among items of clinical laboratory tests (hematology, blood chemistry, urinalysis, and thyroid function, etc.), most of the parameters, except total cholesterol and urine red blood cell (RBC), did not show statistically significant changes after treatment (Table 3). There was significant difference in total cholesterol of treatment group, which was due to change from abnormal to normal (*p* = 0.025). In the urinalysis, there was a significant difference in urine RBC after 52 weeks of treatment within the treatment group and the comparator group (treatment group: *p* = 0.008; comparator group: *p* = 0.034), that was the change from normal status to clinically significant hematuria in two cases of the treatment group All of them were reported as adverse events and judged to be unrelated to the investigational product. In the comparator group, one subject showed increased levels aspartate aminotransferase (AST) and alanine aminotransferase (ALT). They were reported as adverse reactions (AST increased, ALT increased) and judged to be unrelated to the investigational product. These levels were normalized after the study was completed. Some subjects had normal thyroid-stimulating hormone (TSH) levels at baseline, but showed clinically significant abnormal levels after treatment with the investigational product. However, all of them were normal after the study was completed without treatment. All elevations were due to hypothyroidism history and reported as adverse events (blood TSH increased). All were considered to be unrelated to the investigational product. No subject showed a clinically significant abnormality in the free thyroxine (fT4) test at baseline or after treatment with the investigational product. In the antibody test, statistically significant difference from baseline was not found in both the treatment group and the comparator group at 26 weeks and 52 weeks (treatment group at 26 weeks and 52 weeks, *p* = 0.563 and *p* = 0.137, respectively; comparator group at 26 weeks and 52 weeks, *p* = 0.709 and *p* = 0.329, respectively). There was no difference in antibody test results between two groups at any time point (at 26 weeks and 52 weeks, *p* = 0.779 and *p* = 0.742, respectively).

**Table 3:** Laboratory results at baseline and week 52 in the treatment and comparative groups

		Treatment group (*N* = 27)		Comparator (*N* = 31)	
		N	Mean ± SD	N	Mean ± SD
ALT (IU/L)	Baseline	27	18.9 ± 14.4	31	20.4 ± 14.1
	Week 52	26	18.3 ± 19.9	29	19.3 ± 16.9
AST (IU/L)	Baseline	27	30.4 ± 7.5	31	32.3 ± 7.2
	Week 52	26	28.2 ± 10.5	29	30.3 ± 13.0
ALP (IU/L)	Baseline	27	244.6 ± 164.2	31	274.0 ± 178.0
	Week 52	26	298.0 ± 197.3	29	291.1 ± 162.6
Cholesterol (mg/dL)	Baseline	27	173.9 ± 26.8	31	170.7 ± 31.7
	Week 52	26	166.9 ± 21.2	29	160.6 ± 22.5
Triglyceride (mg/dL)	Baseline	27	89.5 ± 61.6	31	119.9 ± 70.3
	Week 52	26	99.5 ± 48.8	29	116.3 ± 89.0
Hemoglobin A1c (%)	Baseline	27	5.01 ± 0.44	31	5.17 ± 0.35
	Week 52	26	5.17 ± 0.45	29	5.32 ± 0.27
TSH (uIU/mL)	Baseline	27	3.06 ± 1.51	31	3.13 ± 1.65
	Week 52	26	2.81 ± 1.62	29	3.14 ± 2.16
Free T4 (ng/ dL)	Baseline	27	1.39 ± 0.27	31	1.37 ± 0.20
	Week 52	26	1.36 ± 0.24	29	1.35 ± 0.18

Among results of vital signs, there were statistically significant changes in systolic blood pressure within the comparator group and differences between the two groups at 13 weeks. However, these changes were temporary and had no clinical significance. There was no statistically significant difference at other time points. In diastolic blood pressure and pulse, there was also no statistically significant differences between the two groups and among different time points within each group.

## Discussion

Growth in girls with TS is characterized by a slight to moderate intrauterine growth restriction, a decreased growth velocity during infancy and childhood, and failure to experience a pubertal growth spurt [14]. GH treatment is standard care for a girl with TS. It is usually started after the age of 2 years as soon as the girl’s height falls below the third to fifth percentile for the population in the same age cohort [6]. GH therapy aims at early catch-up growth in childhood so that normal height could be achieved before puberty [6, 7, 9, 15]. However, more than 20% of girls with TS receive a diagnosis in mid-childhood on investigation of short stature because their clinical presentation of TS such as broad shield like chest with widely spaced nipples, webbed neck, and low hairline can be mild or absent [14, 16, 17]. TS is the most common cause of genetic origin in otherwise healthy girls with a short stature except for those with a familial short stature or constitutional delay of puberty [1, 18]. For girls with a delayed diagnosis of TS, GH therapy is still recommended in most cases. A small gain in height might occur even after the age of 12 or adolescence because of delayed epiphyseal closure due to estrogen deficiency [19].

A study on the efficacy of recombinant human growth hormone in patients with TS was initiated in 1983 in the United States, leading to the approval of this agent by the Food and Drug Administration in 1997 [20]. GH therapy in the United States is generally initiated at FDA-approved dose of 1.125 IU/kg per week (0.375 mg/kg per week) and adapted according to growth velocity and IGF-1 levels of girls with TS [21]. In Korea, the approved dose (1.0–1.4 IU/kg per week) is similar to but slightly higher than that in United states. Several studies have used doses of GH higher than those approved by the FDA, giving a relatively small gain in height with higher IGF-I levels compare to those with the approved dose [22]. Individual responses to GH are variable according to different protocols such as age at start of GH treatment, dosing regimens, consistency of treatment, management of puberty, and adjuvant therapy [11, 12, 23]. GH treatment started in toddler showed early normalization of short stature, however growth failure can be prevented if GH treatment is started before six years of age without interruption [12]. Height and height SDS at the start of treatment are also related to growth response after GH treatment for TS [24–26]. Among them, GH dose is the most important predictor of HV in the first year of GH treatment for TS patients [27]. In the present study, subjects in both groups were administered an approved dose of GH (0.14 IU (0.045–0.050 mg)/kg/day). There were larger increases of HV and HtSDS during one year of GH treatment compared to those in a former Korean study [28]. Increased HV in the first year of GH treatment for girls with TS is the most important factor in predicting the final adult height [27]. In addition, there was no significant difference in the number of poor response subjects according to first-year HV between the present study and a previous study [29]. Previous studies and meta-analysis of beneficial effect of long term GH treatment have shown 5–7 cm and 1.22 HtSDS increase in final adult height (147.8–152.3 cm) [7, 8, 10, 24, 26, 30]. Some reports have indicated an increase of 8 to 10 cm in final adult height after receiving at least 6 years of GH therapy with delayed estrogen administration [20, 25]. However, delayed estrogen administration can be deleterious to bone, uterine, and psychosocial aspects, recent guideline recommend that estrogen treatment should began at 11 or 12 years of age in girls with high gonadotropin or low anti-Mullerian hormone [7, 31]. A recent Korean study has also shown noticeable increase of final adult height by 12 cm after 6 years of treatment, with half of patients having TS attaining normal heights [10]. Ahn et al. have shown that a younger age, a longer duration of GH treatment, and a larger dose can lead to an outstanding increase of final height [10].

Besides growth promotion effect, GH therapy in girls with TS has beneficial effect on lipid profile with cardioprotective value and lower prevalence of arterial hypertension [13, 32]. The present study also showed improved cholesterol without adverse effect on blood pressure, although there was a temporary elevation of systolic blood pressure that was not statistically significant. Women with TS are predisposed to cardiovascular diseases and congenital cardiovascular malformation since risk factors of cardiovascular diseases including hypertension, hyperlipidemia, and insulin resistance are more often found in TS [6]. Echocardiography and MRI studies of GH treated-girls with TS have revealed normal left ventricular function without deleterious effect on aortic diameter or compliance [6]. Although GH therapy has a good safety profile in girls with TS, close monitoring of IGF1 levels, abnormal glucose tolerance, reduced insulin sensitivity, benign intracranial hypertension, scoliosis and other skeletal changes at intervals of 3–6 months-is recommended [6, 7]. Long-term GH therapy has variable effects on skeletal development including craniofacial features such as micrognathia, high-arched palate, short fourth metacarpals, genu valgum, Madelung wrist deformities, and short limbs in TS girls [13, 33, 34]. A recent study has shown high prevalence of retrognathism, skin adnexa abnormality, dense eyebrows, and long lashes with the occurrence of nail anomalies and/or ingrowing nails in girls with TS [13]. A lichen planus-like drug eruption associated with GH therapy has been reported in a child with TS [35, 36].

There is no consistent report on the difference of adult height according to karyotype [4, 37]. Recent French national study showed that growth retardation in TS tends to be more severe in patients with isochromosome or ring chromosome of X and in patients with a karyotype 45, X to a lesser extent than in patients with other karyotypes [37]. Loss of interstitial or terminal long-arm material of the X chromosome (Xq) can result in short stature and primary ovarian failure. In general, loss of the short arm of the X chromosome (Xp) will result in a short stature with typical skeletal changes observed in individuals with TS, in part as a result of haploinsufficiency of the short stature–homeobox (SHOX) gene located in the pseudoautosomal region (PAR1) of Y and Xp [38]. Approximately half patients with TS have a 45,X karyotype while the remaining patients have isochromosome Xq, mosaicism or structural abnormalities of the X chromosome [1, 39]. Contrast to studies of Sybert et al. and Stochholm et al. [1], mosaicism with structural abnormality of X chromosome was highly observed in subjects of the present study. Several studies in Korea have also shown a higher incidence of mosaicism [24]. The fact that mosaicism is often observed in patients with TS might be related to chromosome analysis that is frequently conducted, even for Korean patients with short stature who do not have other characteristic features of TS. This phenomenon occurs because many Korean parents are severely concerned about their children’s current and final heights. The high prevalence of mosaicism in patients with TS might have considerably increased the HV and final height in Korean studies [10]. Moreover, the polymorphism of genes associated with TS might play a role in individual response to GH [40, 41].

## Conclusion

In conclusion, this clinical study demonstrated an excellent growth promoting effect of DA-3002 in children with short stature due to TS and confirmed its safety. Thus, DA-3002 is another effective option for patients with TS considering GH treatment without any safety concern. Early GH treatment with DA-3002 in TS patients is expected to increase HV and improve their final heights, although a long-term study is needed.

## Data Availability

All data generated or analyzed during this study are included in this published article.

## Abbreviations

ADR: Adverse drug reaction
ALP: Alkaline phosphatase
ALT: Alanine aminotransferase
ANCOVA: Analysis of Covariance
AST: Aspartate aminotransferase
BMI: Body mass index
CI: Confidence interval
FA set: Full-analysis set
FDA: Food and Drug Administration
fT4: Free thyroxine
GH: Growth hormone
HbA1c: Haemoglobin A1c
HtSDS: Height standard deviation score
HV: Height velocity
IGF-1: Insulin-like growth factor-1
IGFBP-3: Insulin-like growth factor binding protein-3
PP set: Per-protocol set
RBC: Red blood cell
RCT: Randomized controlled trial
rhGH: Recombinant human GH
SAE: Serious adverse event
SD: Standard deviation
SDS: Standard deviation scores
SE: Standard error
TEAE: Treatment-emergent adverse event
TS: Turner syndrome
TSH: Thyroid stimulating hormone
